# Histamine: a new immunomodulatory player in the neuron-glia crosstalk

**DOI:** 10.3389/fncel.2014.00120

**Published:** 2014-04-30

**Authors:** Sandra M. Rocha, Joel Pires, Marta Esteves, Baltazar Graça, Liliana Bernardino

**Affiliations:** Health Sciences Research Centre, Faculty of Health Sciences, University of Beira InteriorCovilhã, Portugal

**Keywords:** microglia, histamine, nitric oxide, substantia nigra, neuroinflammation, Parkinson’s disease

## Abstract

Histamine is an amine acting as a major peripheral inflammatory mediator. In the brain, histamine was initially viewed as a neurotransmitter, but new evidences support its involvement in the modulation of innate immune responses. Recently, we showed that histamine modulates microglial migration and cytokine release. Its pleiotropic actions, ranging from neurotransmission to inflammation, highlight histamine as a key player in a vast array of brain physiologic activities and also in the pathogenesis of several neurodegenerative diseases. Herein, we emphasize the role of histamine as a modulator of brain immune reactions, either by acting on invading peripheral immune cells and/or on resident microglial cells. We also unveil the putative involvement of histamine in the microglial-neuronal communication. We first show that histamine modulates the release of inflammatory mediators, namely nitric oxide, by microglia cells. Consequently, the microglia secretome released upon histamine stimulation fosters dopaminergic neuronal death. These data may reveal important new pharmacological applications on the use histamine and antihistamines, particularly in the context of Parkinson’s disease.

## Histamine: general aspects

**Figure 1:**
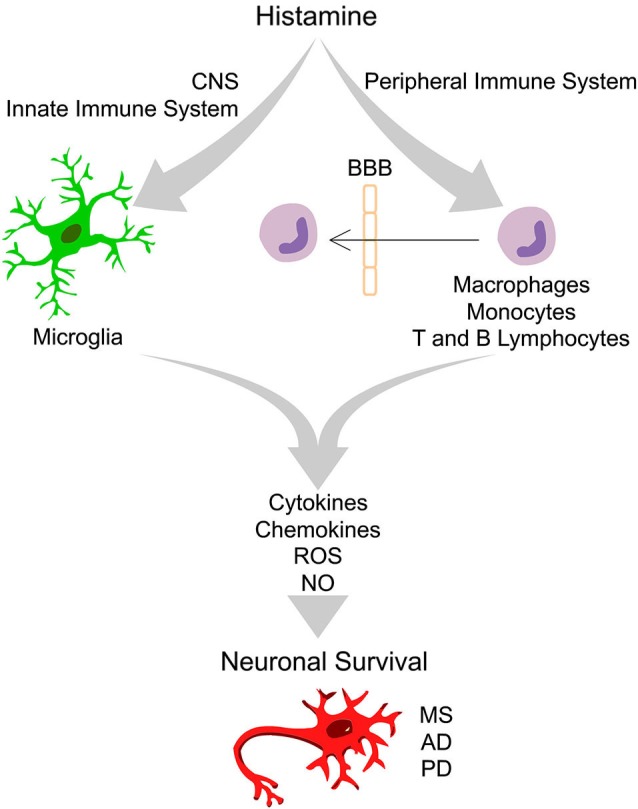
**Integrative scheme of the effects driven by histamine in peripheral and innate immune cells that ultimately may interfere with neuronal survival.** In the healthy brain the “bulk” concentration of histamine is very low. Upon brain injury, degeneration or infection, the inflammatory response may trigger degranulation of mast cells, leading to a massive release of histamine in the blood and in the cerebrospinal fluid, leading to an increase of blood brain barrier (BBB) permeability. In this context, peripheral immune cells may cross the BBB and invade the brain parenchyma. Increased levels of histamine may activate distinct histamine receptors at peripheral (macrophages, monocytes, T and B lymphocytes) and innate immune cells (microglia) leading to the release of pro- or anti-inflammatory cytokines, chemokines, reactive oxygen species (ROS) and nitric oxide (NO). Histamine may thus have a dual role in the modulation of neuronal survival in the context of several neurodegenerative diseases, including Multiple Sclerosis (MS), Alzheimer’s disease (AD) and Parkinson’s disease (PD), depending on the type of histamine receptor activated, signaling pathways involved and factors released.

Histamine is an endogenous biogenic amine synthesized from L-histidine through the catalytic activity of the enzyme histidine decarboxylase. In peripheral tissues, mast cells and basophils are the main sources of histamine. Other sources of histamine include gastric enterochromaffin-like cells, platelets and neutrophils. In the adult brain, histamine is produced by neurons, mast and microglia cells (Katoh et al., 2001; Haas et al., 2008). Histamine exerts its functions through the activation of four distinct receptors belonging to the rhodopsin-like family of G protein-coupled receptors (GPCRs): H_1_ receptor (H_1_R), H_2_ receptor (H_2_R), H_3_ receptor (H_3_R) and H_4_ receptor (H_4_R). These functions range from the modulation of the allergic reactions and behavioral state and reinforcement (H_1_R); regulation of heart and gastric acid secretion, learning and memory (H_2_R); neurotransmitter release, cognition and memory (H_3_R); and chemotactic effects (H_4_R), among others (Haas et al., 2008; Molina-Hernández et al., 2012). Three (H_1_R, H_2_R and H_3_R) out of the four histamine receptors are widely expressed in the nervous system (Haas et al., 2008; Leurs et al., 2009). However, the expression of H_4_R has remained controversial. Several groups could not detect H_4_R mRNA, while other labs reported their expression in the amygdala, cerebellum, hippocampus, caudate nucleus, substantia nigra (SN), thalamus and hypothalamus (Strakhova et al., 2009). Recently, we found that all four types of histamine receptors are expressed by N9 microglia cell line and cortical-derived microglial cells (Ferreira et al., 2012). The levels of histamine and its metabolites have been evaluated in physiologic and pathologic brain conditions. The concentrations of histamine found in the cerebrospinal fluid (CSF) and parenchyma of the intact brain are very low, at the nanomolar range (Croyal et al., 2011). Importantly, circulating levels of histamine and histaminergic innervations are increased following brain injury and degeneration or infection, suggesting that histamine may have an important role in modulating neuronal survival (Anichtchik et al., 2000a; Figure 1).

### Histamine: a peripheral immune mediator

Several brain injuries and inflammatory conditions are associated with increased levels of circulating histamine, both in the blood and in the CSF, leading to blood brain barrier permeability (BBB). Histamine may thus modulate the expression of several inflammatory molecules by peripheral immune cells, including, macrophages, monocytes, T and B lymphocytes (Jutel et al., 2006). In these conditions, activated peripheral immune cells may cross the BBB and exert its immunomodulatory activities in the brain parenchyma (Figure 1).

Histamine may play a dual role in the inflammatory response driven by macrophages. In fact, it was shown that histamine induced interleukin (IL)-6 release by macrophages via H_1_R activation (Marone et al., 2001), whereas inhibited chemotaxis, phagocytosis, Tumor Necrosis Factor (TNF)-α, IL-12 and superoxide anion production via H_2_R (Azuma et al., 2001). Histamine can also stimulate monocytes, but not macrophages, to express monocyte chemoattractant protein (MCP)-1 and its receptors CCR2-A and -B via H_2_R receptor activation (Kimura et al., 2004). Gschwandtner et al. showed that histamine down-regulated IL-27 production by human monocytes through H_2_R and H_4_R, but did not influence IL-6, IL-10 and TNF-α production (Gschwandtner et al., 2012). Lymphocytes are predominantly involved in adaptive immunity and play a key role in the pathogenesis of brain disorders associated with inflammation, such as multiple sclerosis (MS). T cells express histamine receptors being thus responsive to histamine stimulation. For instance, histamine enhances T_H_1-type activity through H_1_R, whereas both T_H_1- and T_H_2-type responses are negatively regulated by H_2_R (Jutel et al., 2001). The impact of histamine on immunoglobulin secretion by B cells depends on the requirement of T cells (Ferstl et al., 2012).

In conclusion, the effects of histamine on peripheral immune cells are often contradictory leading either to the stimulation or inhibition of inflammatory processes. This can be due to the type, affinity and abundance of each receptor subtype and consequently to the involvement of distinct downstream regulatory pathways, to the levels of histamine and to the cell types used in each study. Even if comprehension regarding the regulatory pathways activated by each histamine receptor is warranted, there are already several evidences that strengthen the idea that histamine and its receptor agonists/antagonists are promising targets to modulate inflammatory conditions.

### Histamine: new modulator of microglial activity

Microglial cells, the resident immune cells in the brain, have the ability to patrol and protect the parenchyma against brain injuries and/or infections. In a healthy environment, resting microglia display low expression levels of inflammatory molecules, but when activated, microglial cells abandon their ramified surveilling morphology, become ameboid, phagocytic, migrate to the injured site and release inflammatory molecules (Polazzi and Monti, 2010). The density and phenotype of microglial cells are region-specific. In particular, a higher density of microglia in the substantia nigra pars compact (SNpc) lead several authors to suggest that microglia play an important role in the pathogenesis of Parkinson’s disease (PD; Collins et al., 2012). In accordance, higher numbers of activated microglia were found in the vicinity of degenerating SNpc dopaminergic neurons of post-mortem PD brains (Long-Smith et al., 2009). A major unanswered question is whether microglia activation is a consequence or a cause of nigra dopaminergic cell loss. Recent studies suggest that neuronal loss leads to the extracellular release of protein aggregates from neurons causing microglia activation (Hafner-Bratkovič et al., 2012). On the other hand, a large body of literature also supports the idea that microglia potentially increase the risk of development and exacerbation of the neurodegenerative pathology (Phani et al., 2012).

**Figure 2:**
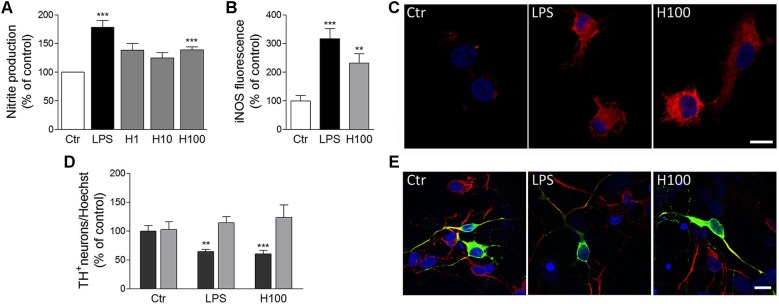
**Histamine induced NO release by microglial cells and subsequent dopaminergic neuronal death. (A)** Histamine at 1 μM (H1), 10 μM (H10) and 100 μM (H100) triggered an increase of NO release by microglial cells. LPS (100 ng/ml) was used as a positive control. **(B)** Bargram shows that histamine increased inducible nitric oxide synthase (iNOS) expression by microglial cells. **(C)** Representative fluorescent digital images of microglial cell cultures treated with 100 μM histamine or 100 ng/ml LPS and stained against iNOS (red staining). For nuclear labeling, cells preparations were counterstained with Hoechst (2 μg/mL; blue staining). **(D)** The conditioned medium derived from microglial cells pre-treated with 100 μM histamine or 100 ng/mL LPS decreased the numbers of TH^+^-neurons (dark gray bars). The conditioned medium pre-treated solely with histamine or LPS (devoid of microglia-induced soluble factors) did not affect dopaminergic neuronal survival (light gray bars). Results are expressed as the mean value of TH^+^ cells in relation to all nuclei stained with Hoechst. **(E)** Representative fluorescence digital images of midbrain neuronal-glial co-cultures treated with microglia-derived conditioned medium. Green staining: TH^+^ neurons; red staining: MAP-2 positive neurons; blue staining: nuclei. Scale bar = 10 μm. Ctr: control; LPS: 100 ng/mL LPS; H100: 100 μM histamine. Data are expressed as mean ± SEM. Statistical analysis was performed using one-way ANOVA with Dunnett’s correction. ** *P* < 0.01 and *** *P* < 0.001 as compared with the untreated control—set to 100%.

Microglia release a huge number of inflammatory mediators, including histamine (Katoh et al., 2001). We have recently shown, for the first time, that histamine modulates microglial migration and cytokine release (Ferreira et al., 2012). Histamine, acting via H_4_R, increased microglial motility through the involvement of α5β1 integrins and the p38 MAPK and Akt signaling pathways. Additionally, histamine also modulated IL-1β release by microglia (Ferreira et al., 2012). In that sense, histamine may have an impact in the pathogenesis of brain diseases which are associated with inflammatory conditions (Figure 1). Our previous study was performed using a N9 microglia cell line, but there is a lack of information regarding the effects of histamine in microglia cells derived from the SN, a brain region enriched with microglia and highly susceptible to dopaminergic neuronal loss. Thus, we evaluated the effects of histamine on the production of an inflammatory mediator, nitric oxide (NO), by microglial cells derived from the SN of neonatal rats. We measured the amount of nitrite (a stable metabolite of NO) released by primary microglial cells after 24 h of treatment with different histamine concentrations (1 μM; 10 μM and 100 μM). Lipopolysaccharide (LPS; 100 ng/mL), a potent stimulator of microglia activation that causes the release of various inflammatory factors and free radicals, was used as a positive control. Microglial cells, isolated from the ventral midbrain of postnatal (P2-3) Wistar rats, were grown in Dulbecco’s modified Eagle’s medium (DMEM) supplemented with 10% Fetal Bovine Serum and 100 U/mL penicillin plus 100 μg/mL streptomycin, as described previously (Saura et al., 2003). The Griess Assay was used to measure NO production and values were expressed as percentage of increase with respect to the untreated cultures (controls set to 100%). We found that histamine significantly increased NO release (mean_H1_ = 138.5 ± 11.7; mean_H10_ = 125.0 ± 9.4; mean_H100_ = 140.2 ± 5.1, *n* = 3–15), as compared to control (Figure 2A). Histamine did not interfere with microglia cell death or proliferation at all concentrations tested (data not shown). As expected, 100 ng/ml LPS increased NO production (mean_LPS_ = 178.4 ± 12.2, *n* = 16; Figure 2A). Based on these results and on prior studies reported by our group (Agasse et al., 2008; Bernardino et al., 2008; Grade et al., 2010; Rosa et al., 2010) and by others (Wang et al., 1997; Hernández-Angeles et al., 2001; Nicolson et al., 2002; Tran et al., 2004; Molina-Hernández and Velasco, 2008; Nemeth et al., 2012), we then used 100 μM histamine in further experiments, a concentration of pathophysiological relevance.

NO is produced from L-arginine by different isoforms of NOS and takes part in many normal physiological functions, such as promoting vasodilation of blood vessels and mediating cell communication within the brain. In addition to its physiological actions, the free radical activity of NO may cause cellular damage through a phenomenon known as nitrosative stress (Knott and Bossy-Wetzel, 2009). Since the main inducible enzyme responsible for NO synthesis in microglia cells is inducible nitric oxide synthase (iNOS), we then hypothesized that iNOS expression was also upregulated by histamine. To test this hypothesis, microglial cells were treated for 24 h with 100 ng/mL LPS or 100 μM histamine, fixed and stained against iNOS (polyclonal rabbit anti-iNOS; 1:100; BD Transduction Laboratories). Fluorescent images were acquired using a Zeiss inverted microscope (Axiobserver Z1, Zeiss) and the fluorescence intensity was measured through ImageJ software (60 cells *per* condition). The background fluorescence intensity was always subtracted in order to quantify the corrected intensity of the iNOS fluorescence in each condition. The same confocal image acquisition settings were used in all experiments. As shown the Figures 2B and 2C, both histamine and LPS significantly increased the expression of iNOS in microglial cells (mean_H100_ = 232.0 ± 31.8; mean_LPS_ = 316.6 ± 36; *n* = 3). This suggests that histamine could act as a NO-regulating factor by inducing iNOS expression. Others also showed that histamine stimulates endothelial NO production and iNOS expression via H_1_R and nuclear factor (NF)-kappaB signaling pathway in intimal smooth muscle cells (Tanimoto et al., 2007). NO has been shown to modify protein function by nitrosylation and nitrotyrosination, to contribute to glutamate excitotoxicity, inhibit mitochondrial respiratory complexes, participate in organelle fragmentation, and mobilize zinc from internal stores (Knott and Bossy-Wetzel, 2009). NO can react with superoxide radicals to form peroxynitrite radicals that are short-lived oxidants and highly damaging to neurons. Mitochondrial injury is prevented by treatment with an iNOS inhibitor, suggesting that iNOS-derived NO is also associated with the mitochondrial impairment (Choi et al., 2009). NO inhibits cytochrome c oxidase in competition with oxygen, resulting in glutamate release and excitotoxicity (Brown and Neher, 2010). However, the molecular mechanisms involved on the modulatory effect of histamine on NO production are unknown. Nevertheless, it is known that activation of extracellular signal-regulated kinases, c-Jun N-terminal kinases and p38 MAPK leads to activation of transcription factors, such as NF-kappaB and activator protein 1 (AP-1), that are involved in expression of the iNOS gene, which is followed by the sustained production of NO by activated microglia (Jung et al., 2008). Since LPS-induced NO production by microglia involves all these intracellular signaling pathways, we hypothesize that histamine may also mediate NO production by some of these pathways. The use of selective inhibitors of these pathways as well as the evaluation of their expression and activation may be helpful to disclose the signaling pathways involved in NO-mediated histamine effects.

### Histamine: dual role in neuroprotection/neurodegeneration

The neuronal histaminergic system is involved in many physiological functions and consequently severely affected in age-related neurodegenerative diseases. The production of neuronal histamine shows diurnal fluctuations in control patients who had no neuropsychiatric disorders, while this fluctuation was strongly altered in patients with neurodegenerative diseases (Shan et al., 2013). Moreover, altered levels of histamine found in the diseased brain and in the peripheral system may modulate both the innate and adaptive immune responses ultimately affecting neuronal survival. Brain diseases in which the histaminergic system may be involved include MS, PD, Alzheimer’s disease (AD), among others (Figure 1).

MS is characterized by focal lymphocytic infiltrations to the brain leading to damage of myelin and axons. Initially, the inflammatory response is transient and remyelination occurs, but over time widespread microglial activation ensues along with extensive and chronic neurodegeneration (Passani and Ballerini, 2012). The observation that histamine may be implicated in MS dates back to the early 1980s when Tuomisto et al. reported that patients with remitting or progressive disease showed histamine levels about 60% higher than controls, suggesting an altered histamine metabolism in MS (Tuomisto et al., 1983). However, another clinical study did not show elevated concentrations of histamine and its metabolite methylhistamine in MS patients when compared, in this case, with individuals affected by other neurological diseases (Rozniecki et al., 1995). More recently, gene-microarray analysis has shown that H_1_R expression is upregulated in MS lesions (Lock et al., 2002), and epidemiological studies suggest a protective effect of brain penetrating H_1_R antagonists (Alonso et al., 2006). Furthermore, in a small pilot study, a cohort of MS patients treated with an H_1_R antagonist showed signs of neurological amelioration (Logothetis et al., 2005). The H_1_R has long been associated with inflammatory responses and H_1_R antagonists may be used in brain repair therapies.

The role histamine in AD is also well documented. However, contradictory data exists regarding the quantification of histamine levels in brain compartments of AD patients, making difficult a direct correlation between histaminergic neurotransmission and AD pathology (Brioni et al., 2011). Increased histamine levels have been reported not only in the frontal cortex, basal ganglia and hippocampus, but also, together with its metabolites, in the CSF and serum of AD patients (Fernandez-Novoa and Cacabelos, 2001). On the contrary to the above findings, several other studies show decline in histamine levels in AD brains. Recent studies show that H_3_R antagonists may be efficient in AD therapy. Nathan et al. demonstrated that GSK239512, a selective H_3_R antagonist, displayed a satisfactory level of tolerability in AD patients with evidence of positive effects on attention and memory (Nathan et al., 2013). Others studies also support that H_3_R antagonists/inverse agonists are potentially novel therapeutic targets for other diseases, such as vigilance and sleep-wake disorders (Parmentier et al., 2007) and schizophrenia (Vohora and Bhowmik, 2012).

Several abnormalities in the histaminergic system were also found in PD patients. In post-mortem brain of PD patients, it has been reported a dramatic increase of histaminergic innervations, enlarged axonal innervations (Anichtchik et al., 2000a) and increased histamine levels (Nuutinen and Panula, 2011), while animal studies showed that increased endogenous histamine levels may accelerate degeneration of SN dopaminergic neurons in 6-hydroxydopamine (OHDA) lesioned rats (Liu et al., 2007). In addition, increased mRNA levels of histamine methyltransferase (HMT), a key enzyme involved in histamine metabolism, were found in the SN and in the putamen of PD patients (Shan et al., 2012). This increase may act as a protective mechanism by metabolizing enhanced histamine levels in these brain regions. Moreover, a Thr105Ile polymorphism of HMT was shown to be associated with PD (Palada et al., 2012). Regarding the expression of histamine receptors, it has been shown that H_3_R mRNA was significantly decreased in the SN, while H_4_R mRNA expression showed a significant increase in caudate nucleus and putamen in PD patients (Shan et al., 2012). Vizuete et al. showed that the SN dopaminergic neurons are highly sensitive to histamine-induced neurotoxicity (Vizuete et al., 2000). Altogether, these data suggest that histamine and its receptors may play an important role in PD pathogenesis. However, it is still unclear how microglia activation induced by histamine may contribute to dopaminergic neuronal survival. Thus, we aimed to uncover whether soluble factors released by microglia previously stimulated with histamine or LPS could modulate dopaminergic neuronal survival. LPS *per se* does not seem to have a direct effect on neuronal viability making it an excellent tool to study inflammation-mediated dopaminergic neurodegeneration (Block et al., 2007). In our study, dopaminergic neuronal viability was assessed by counting the numbers of tyrosine hydroxylase (TH^+^)-immunoreactive neurons (using monoclonal mouse anti-TH; 1:100 dilution; Transduction Laboratories), counterstained with MAP2 (using polyclonal rabbit anti-MAP2; 1:200 dilution; Santa Cruz Biotechnologies), on neuron-astrocyte midbrain co-cultures from neonatal rats. The midbrain cultures were obtained from Wistar pregnant females with 15–16 gestational days and were grown in Neurobasal medium supplemented with 2% B27, 25 μM/mL glutamate, 0.5/mL glutamine and 120 μg/mL gentamicine for 5–6 days as described in Campos et al. (2012). Conditioned medium derived from untreated microglial cells were considered as the control condition and the resulting values were set to 100%. As shown in Figures 2D and 2E, the conditioned media derived from microglial cells pre-treated for 24 h with 100 μM histamine or 100 ng/mL LPS induced a 30% reduction in the number of TH^+^ cells (mean_H100_ = 69.2 ± 4.3; mean_LPS_ = 68.5 ± 1.5; *n* = 4; dark gray bars). To ensure that this effect was solely dependent on soluble factors released by microglial cells, we then incubated midbrain neuronal cultures with culture media pre-treated for 24 h with LPS or histamine, at 37°C, but in the absence of microglial cells. We found that the dopaminergic neuronal survival was not affected when midbrain neuronal cultures were treated with conditioned medium devoid of microglia-released factors (mean_H100_ = 123.2 ± 28.5; mean_LPS_ = 115.5 ± 18.4; *n* = 3; Figure 2D; light gray bars). Herein we showed, for the first time, that histamine promotes the release of toxic inflammatory factors, including NO, by microglial cells, which can be capable of damaging dopaminergic neurons. With this work we open a new perspective for the therapeutic use of histamine and histamine receptor antagonists to treat or ameliorate inflammation-associated processes of neurodegenerative diseases, like those seen in PD.

## Future perspectives

Accumulating clinical and experimental evidences show that changes in the histaminergic system may be associated with the pathogenesis and progression of several neurodegenerative diseases, including PD. We showed that histamine boosts neuroinflammation by promoting microglia migration and the release of cytokines and NO. We also showed that production of neurotoxic and inflammatory mediators by microglial cells upon histamine stimulation leads to dopaminergic neurodegeneration. Based on these data we may infer that microglia activation induced by histamine may contribute to PD pathology, and may thus provide a rationale for possible novel therapeutic strategies. We suggest that the therapeutic use of histamine receptors antagonists may be of great value to treat or ameliorate CNS pathologies or neurodegenerative disorders which are commonly accompanied by inflammation. Therefore, the following steps urge a better understanding of the involvement of histamine and its receptors in the modulation of microglial activation and subsequent neuronal survival.

## Author contributions

Sandra M. Rocha: Provision of study material; Collection and assembly of data; Data analysis and interpretation; Manuscript writing.

Joel Pires: Provision of study material; Collection and assembly of data; Data analysis and interpretation.

Marta Esteves: Provision of study material; Data analysis and interpretation.

Graça Baltazar: Conception and design; Provision of study material; Critical reading of manuscript.

Liliana Bernardino: Conception and design; Provision of study material; Data analysis and interpretation; Administrative support; Financial support; Manuscript writing; Final approval of manuscript.

## Conflict of interest statement

The authors declare that the research was conducted in the absence of any commercial or financial relationships that could be construed as a potential conflict of interest.
